# Improved Homology Model of the Human *all*-trans Retinoic Acid Metabolizing Enzyme CYP26A1

**DOI:** 10.3390/molecules21030351

**Published:** 2016-03-15

**Authors:** Mohamed K. A. Awadalla, Thamir M. Alshammari, Leif A. Eriksson, Patricia Saenz-Méndez

**Affiliations:** 1 Department of Pharmacology and Toxicology, College of Pharmacy, University of Hail, P. O. Box 2440, 81451 Hail, Saudi Arabia; m.awadalla@uoh.edu.sa; 2 Department of Clinical Pharmacology, College of Pharmacy, University of Hail, P. O. Box 2440, 81451 Hail, Saudi Arabia; th.alshammari@uoh.edu.sa; 3 Department of Chemistry and Molecular Biology, University of Gothenburg, 40530 Gothenburg, Sweden; 4 Computational Chemistry and Biology Group, Facultad de Química, UdelaR, 11800 Montevideo, Uruguay; psaenz@fq.edu.uy

**Keywords:** CYP26A1, homology model, RAMBA, retinoic acid, molecular docking

## Abstract

A new CYP26A1 homology model was built based on the crystal structure of cyanobacterial CYP120A1. The model quality was examined for stereochemical accuracy, folding reliability, and absolute quality using a variety of different bioinformatics tools. Furthermore, the docking capabilities of the model were assessed by docking of the natural substrate all-*trans*-retinoic acid (*at*RA), and a group of known azole- and tetralone-based CYP26A1 inhibitors. The preferred binding pose of *at*RA suggests the (4*S*)-OH-*at*RA metabolite production, in agreement with recently available experimental data. The distances between the ligands and the heme group iron of the enzyme are in agreement with corresponding distances obtained for substrates and azole inhibitors for other cytochrome systems. The calculated theoretical binding energies agree with recently reported experimental data and show that the model is capable of discriminating between natural substrate, strong inhibitors (R116010 and R115866), and weak inhibitors (liarozole, fluconazole, tetralone derivatives).

## 1. Introduction

Retinoic acid (RA) is the most active metabolite of vitamin A (retinol), which mediates the biological functions of the vitamin. RA plays an important role in regulating embryonic development, maintenance of adult epithelial tissues, as well as controlling processes including immune response, cell proliferation and differentiation, and apoptosis [1]. Upon RA binding to the nuclear retinoic acid receptor (RAR), RAR heterodimerizes with the nuclear retinoid X receptor (RXR). The resulting dimer interacts with specific DNA retinoic acid response element (RARE) located in the promoter regions of RA target genes [2]. RA is intracellularly synthesized from retinol via two oxidation steps involving oxidation of retinol to retinal and further oxidation to RA by a group of retinol and retinal dehydrogenases, respectively. The endogenously synthesized RA is chemically found in different geometric isomers including 9-*cis*-RA, 11-*cis*-RA, 13-*cis*-RA, (9*Z*,13*Z*)-RA, and the chemically most stable structure *all-trans* RA (*at*RA) [3]. When *at*RA is synthesized, it either binds to the nuclear receptor or undergoes degradation to more polar derivatives by a group of cytochrome P450 oxidase (CYP) enzymes of which CYP1A1, CYP3A4/5/7, CYP4A11 and CYP2C8/9/18 have all been reported to metabolize *at*RA [4,5]. However, the principal family responsible for RA degradation is CYP26.

In humans, the CYP26 family consists of three isoforms: CYP26A1, CYP26B1, and CYP26C1, all capable of recognizing and metabolizing RA as the natural substrate [6,7,8]. Expression pattern studies in human tissues have shown that CYP26A1 is the dominant isoform in the liver [9,10]. *In vivo* and *in vitro* studies have shown that CYP26A1 and B1 are specific for *at*RA, whereas CYP26C1 only metabolizes 9-*cis*-RA efficiently [6,8,9,11]. The resulting *at*RA metabolites include hydroxy, oxo, and epoxy derivatives, of which 4-OH-*at*RA was found to be the main metabolite [4,5]. Since the liver is the principal organ involved in the degradation of endogenous and xenobiotic substances, it is suggested that CYP26A1 plays a major protective role against excessive *at*RA exposure in the body or at least in this organ [9].

Clinically, *at*RA treatment jointly with chemotherapy is the standard approach employed for treating acute promyelocytic leukemia (APL). It is also under investigation for clinical use in other types of cancer and has been indicated to be efficient in treatment of certain dermatological diseases [12,13]. The development of resistance to *at*RA by auto-induction of *at*RA metabolizing enzymes and the narrow therapeutic window of *at*RA are challenging clinical problems [14]. Auto-induction of *at*RA catabolism was found to be caused by CYP26 enzyme overexpression [15,16]. To overcome the auto-induction problem, it was suggested to develop CYP26 inhibitors, known also as RA metabolism blocking agents (RAMBAs). This approach is interesting in particular for the inhibition of the hepatic CYP26A1, which is suggested to be a primary target in the development of new RAMBAs [14,17]. The fundamental principle behind the development of RAMBAs is to increase endogenous *at*RA concentrations in the presence of a CYP26A1 inhibitor, thus potentiating the activity of endogenous *at*RA in a cell-type specific approach [14,18]. This will reduce systemic side effects related to administration of exogeneous *at*RA, often in high doses, and is in agreement with a more targeted therapy [14,18]. Several azole compounds were developed and have been biologically evaluated for CYP26 inhibition. These compounds include talarozole (also known as R115866) [19], R116010 [20], and liarozole [21], but none of them is approved for clinical use in cancer or major dermatological diseases. Liarozole has however been approved with orphan drug status for congenital ichthyosis in Europe and USA [22,23]. Several new classes of compounds have been reported to possess promising inhibitory activity against CYP26A1, including tetralone derivatives and imidazole propanoates, among others [24,25,26].

The absence of a three-dimensional structure of the CYP26A1 enzyme renders rational drug design of new, improved and selective RAMBAs a challenging task. Therefore, the successful use of homology modeling to determine the 3D structure of the CYP26A1 enzyme relies on the availability of a suitable template. In this work, a new CYP26A1 homology model based on the retinoic acid bound cyanobacterial CYP120A1 crystal structure (PDB code 2VE3) [27] has been built and evaluated using different bioinformatics tools and by performing docking studies of tetralone derivatives and azoles reported in recent work [28].

## 2. Results and Discussion

### 2.1. CYP26A1 Homology Model

Previously, five CYP26A1 homology models have been presented in enzyme inhibitor docking studies [29,30,31,32,33]. Human CYP3A4 (PDB code 1TQN) and *Bacillus megaterium* CYP102 (PDB code 1BU7) were used as single templates to build two of the models [30,31]. The third model was obtained in our previous report where the crystal structures of the human CYP2C8 (PDB code 1PQ2), CYP2C9 (PDB code 1OG2) and CYP3A4 (PDB code 1TQN) were used as multiple templates [29]. The templates shared 22% to 26% sequence identity with CYP26A1. This similarity is, however, low and within the “twilight zone”, and may thus not generate reliable homology models [34]. In a previous work, we reported homology models of human CYP26B1 and CYP26B1 spliced variant derived from a more recently reported cyanobacterial CYP120A1 crystal structure (PDB code 2VE3) [35]. In the same work, the use of other templates failed to produce a CYP26B1 spliced variant model capable of accommodating the heme group within the active site. From a BLAST search [36] with solved PDB structures, we found that CYP26A1 shares 33% sequence identity with 2VE3 which is the highest score to a known structure. It has previously been concluded that a protein amino acid sequence with over 30% identity to a known structure often can be modeled with an accuracy comparable to those of medium to low resolution X-ray structures [37]. The fourth model of CYP26A1 previously discussed was built using 2VE3 as template [33]. However, in this model only 87.9% of the residues are located in the favored region after MD simulation and minimization. Moreover, the model contains some important residues in the outlier region of the Ramachandran plot. Finally, during the preparation of this manuscript, a fifth model was also reported, built employing 2VE3 and CYP51A (PDB code 3JUS) as multiple templates [32]. In this model, the Ramachandran plot showed that 99.2% residues are in the favorable or generously allowed regions. Unfortunately, the percentage of residues in the favoured region is not specified, and from the reported Ramachandran plot several (up to twelve) important residues are found in the outlier region. No further quality assessment was discussed in this last report. Although the fourth and fifth models were obtained from the structure with the highest sequence identity (2VE3), the fourth model was not assessed by docking of inhibitors, and the fifth model was not refined nor the reliability of the docked inhibitor poses assessed. This is a problem since it is well known that even though docking of inhibitors is successful with respect to binding pose predictions, the ensuing binding affinities can often be poorly predicted [38,39].

**Figure 1:**
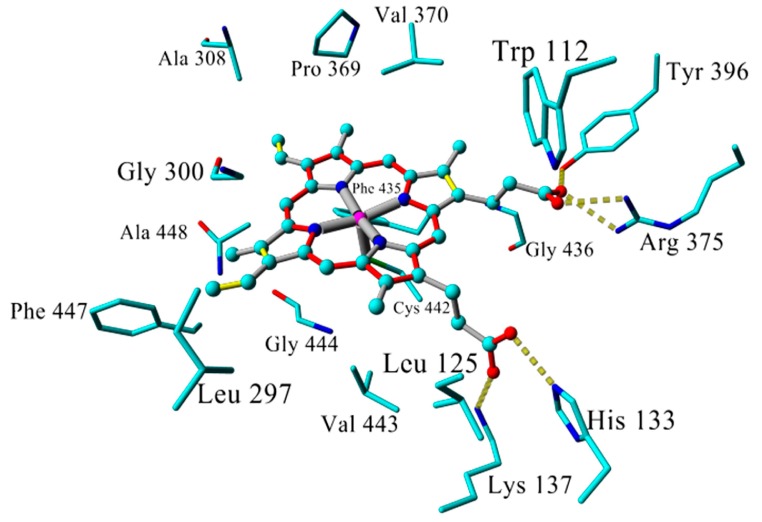
Active site of MD simulated CYP26A1. Heme group in ball and stick, key residues interacting with heme are presented in stick model, and hydrogen bonds are shown as yellow dashed lines. For the heme group, single and double bonds are represented by grey and yellow lines, respectively; resonance bonds by red lines and Fe coordination bonds are represented by grey thick lines. Hydrogen atoms are omitted for clarity.

The preceding discussion triggered the idea of constructing a new high quality CYP26A1 homology model based on the 2VE3 template structure, refining the generated model using MD simulations, and docking of inhibitors on the resulting receptor structure. The model was built and further refined by energy minimization followed by MD simulation. The RMSD values of the position of the alpha carbon atoms over time are shown on Figure S1 (Supplementary Materials). In the model, the heme group showed several hydrophobic interactions with the active site residues including Trp112, Leu125, Leu297, Gly300, Ala308, Pro369, Val370, Phe435, Gly436, Val443, Gly444, Phe447, and Ala448. The heme carboxyl groups formed hydrogen bonds with the side chains of His133, Lys137, Arg375, and Tyr396. The sulfide group of Cys442 occupied the fifth coordination position of the heme iron atom. All interactions orchestrate a perfect placement of the heme group within the active site (Figure 1).

### 2.2. Model Quality

**Figure 2:**
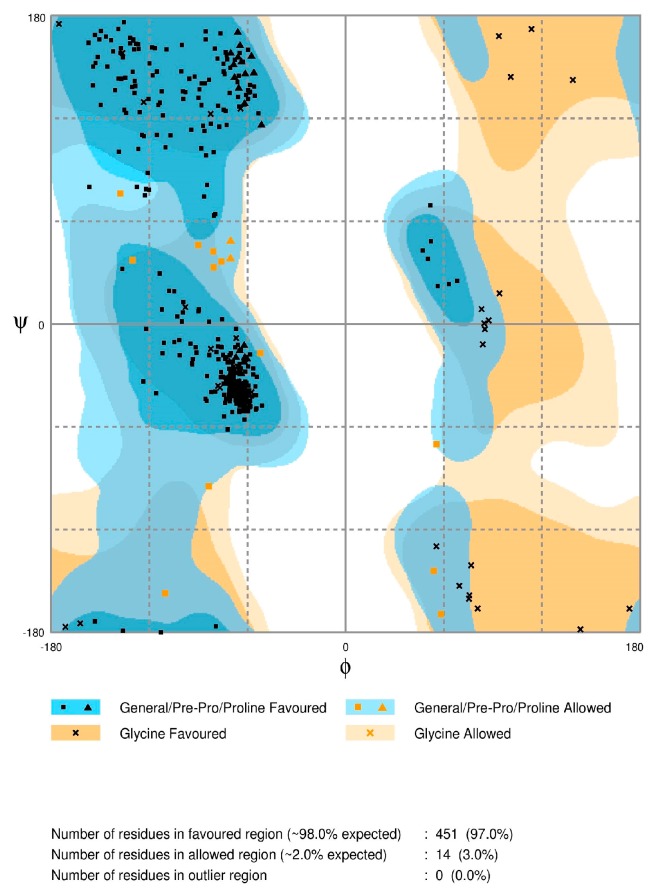
Ramachandran plot of the φ and ψ angles of the current CYP26A1 model.

From RAMPAGE server analysis of the current CYP26A1 model, 97.0% of the residues were found to be located in the favored regions of the Ramachandran plot (Figure 2). Fourteen residues (3%) fell in the allowed regions and no residue was located in the outlier zone. This is in contrast to the Ramachandran plots of the previous models where such data was reported, in which the percentages of residues in the favored regions were 82.9 [29], 82.4 [30], 83.9 [31], and 87.9 [33]. The distribution of the residues in the plot implies that the stereochemical accuracy of the current model is highly satisfactory and has been improved over previously reported models.

**Figure 3:**
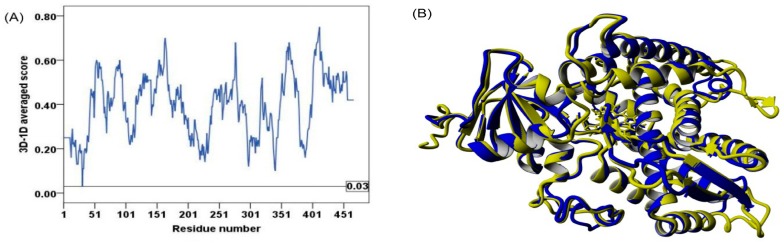
CYP26A1 folding assessment. (**A**) Verify 3D averaged score for the CYP26A1 residues; (**B**) Superposition of CYP26A1 model (blue) and template 2VE3 (yellow).

Verify3D-1D distinguishes between correct and incorrect folding based on a comparison of the 3D amino acid environment of a protein or model to its 1D amino acid sequence. Scores below zero indicate serious folding problems. From the Verify 3D-1D plot (Figure 3A); it is evident that all residues have positive score, with minimum value of 0.03. Furthermore, 92% of the residues have scored ≥0.2 in the 3D-1D profile which is significantly higher than the 80% required for “passing” the test. The result also indicates that the model has an acceptable folding reliability. The overall structural similarity between the refined model and the template is shown as backbone superposition (Figure 3B). The rmsd (root-mean-square deviation) of the superposed structures was 0.75 Å which falls within the acceptable range and indicates high structural template—model similarity and no significant structural drift of the CYP26A1 structure from the template.

**Figure 4:**
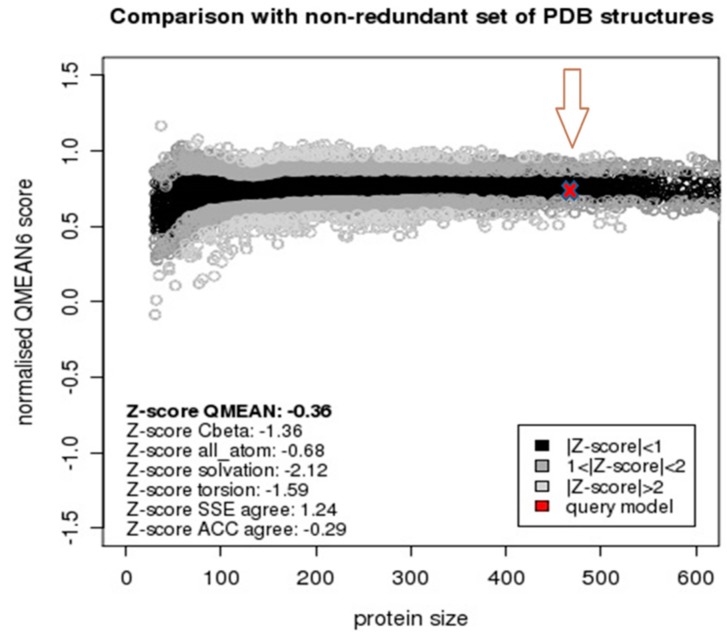
Normalized QMEAN6 z-score of CYP26A1. SSE agree: secondary structure element agreement, ACC agree: solvent accessibility agreement. The arrow is inserted to highlight the position of the query model (red “x”).

The QMEAN server calculates the absolute quality of a model by combining six structural descriptors of the model with respect to scores of a non-redundant set of high resolution X-ray crystallography structures. The normalized QMEAN6 z-score for the model was within the standard deviation value typically obtained for native proteins of similar size (Figure 4), and implies that the model absolute quality is in par with those of already solved structures. Therefore, all results are consistent and acceptable and it can be concluded that the model is of good quality and an improvement over previously presented CYP26A1 models.

### 2.3. Docking

**Figure 5:**
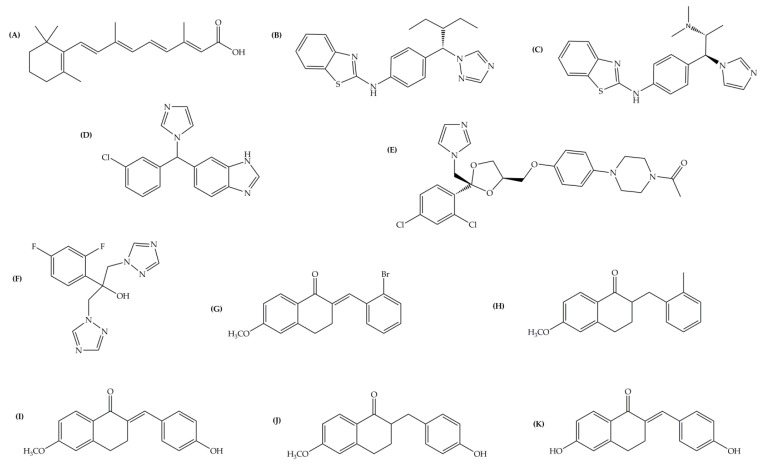
Ligands used in evaluating docking capabilities of the new CYP26A1 model. (**A**) *at*RA; (**B**) R115866; (**C**) R116010; (**D**) liarozole; (**E**) ketoconazole; (**F**) fluconazole; and (**G**) to (**K**) tetralones 1 to 5, respectively.

The model quality was further assessed by investigating its capability towards small molecule docking. To this end, the natural substrate (*at*RA) and known azoles and tetralone based CYP26A1 inhibitors reported in the literature were docked into the model active site (Figure 5).

The top ranked docking poses based on the best Autodock binding energies were selected for further work. Docking of ketoconazole did not generate any pose within the enzyme active site and was therefore excluded from further consideration. In other solved structures of ketoconazole bound to human CYPs such as CYP3A4 (PDB code 2V0M) [40], ketoconacozole has shown atypical enzyme binding where two ketoconazole molecules occupy the binding site, which implies that the enzyme undergoes specific conformational modifications to accommodate the two molecules. This atypical binding behavior may explain the unsuccessful docking of ketoconazole into CYP26A1.

**Figure 6:**
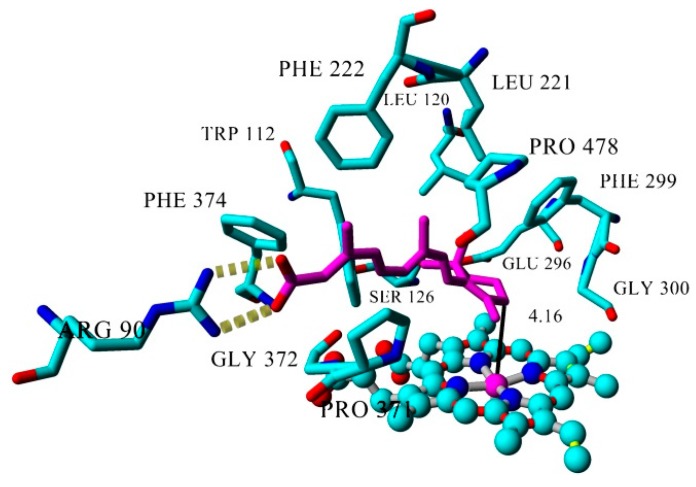
Key interaction residues of *at*RA in the CYP26A1 active site (top ranked structure from Autodock). *at*RA carbon atoms are colored in magenta. Distance between *at*RA C4 and heme Fe is displayed with a continuous black line. Yellow dashed lines represent hydrogen bonds.

All analyses of the ligand-CYP26A1 complexes reported below were performed after energy minimization, starting from the top ranked structure obtained with Autodock. *at*RA docked into the active site of CYP26A1 located the C4 of the former at a distance of 4.16 Å from the heme iron. The hydrophobic cyclohexene and the diterpene moiety of *at*RA allowed the molecule to present hydrophobic interactions with the residues Trp112, Leu120, Ser126, Leu221, Phe222, Glu296, Phe299, Gly300, Val370, Pro371, Gly372, Phe374, Pro498 (Figure 6).

Hydrogen bonds between the *at*RA carboxylic acid tail and Arg90 hold the molecule in place. The orientation of the *at*RA C4 atom towards the heme iron explains the metabolic activity and suggests the formation of a stereoselective metabolite (4*S*)-OH-*at*RA—which is in full agreement with recent experimental findings [33]. The C4—iron distance of 4.16 Å is a reasonable distance for a CYP catalyzed hydroxylation reaction, and also is in agreement with distances obtained from other cytochrome-substrate systems [41].

Although the most recent model was obtained from the same template (2VE3), docking of *at*RA into that model positioned *at*RA C4 atom distant from the heme iron while C2 was closer and in perpendicular position to the heme molecule directly over the heme iron [32]. This docking position suggests C2 as the site of oxidation similar to what is observed in the resolved *at*RA-CYP120A1 complex (2VE3). The prokaryotic enzyme CYP120A1 produces 2-OH-*at*RA as the main metabolite while the main metabolite of the human homolog is 4-OH-*at*RA [4,5,27]. The improved prediction of the docking pose for *at*RA in this work may be attributed to a more reliable homology model as well as to a more accurate molecular docking scheme. As already mentioned, no further assessment was discussed in the previous model [32].

**Figure 7:**
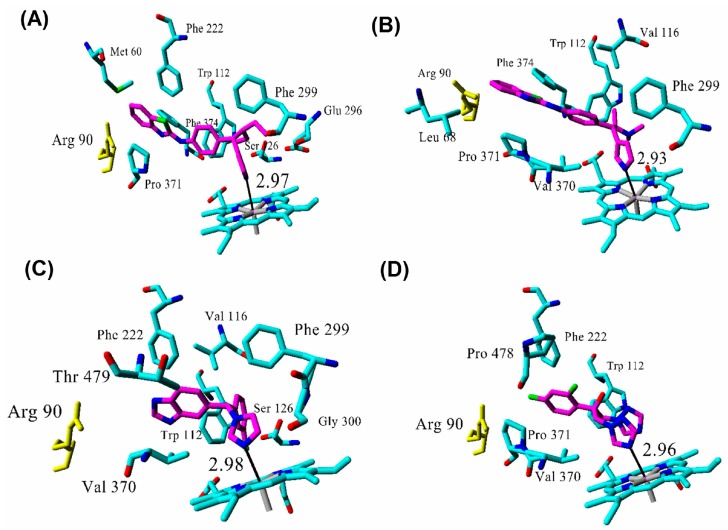
Key residues involved in azole interactions in the CYP26A1 active site (top ranked structures from Autodock). Ligand carbon atoms are colored in magenta. (**A**) R115866; (**B**) R116010; (**C**) liarozole; (**D**) fluconazole. The azole nitrogen—heme iron distances are in angstroms. Arg90 is colored yellow for orientation purposes only.

**Figure 8:**
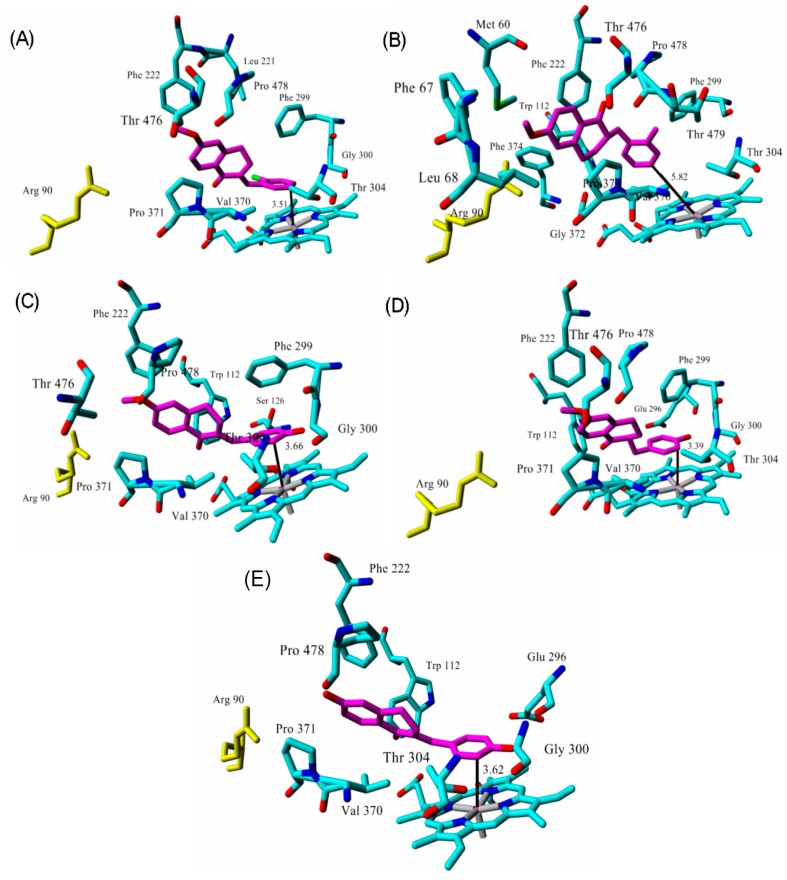
Key residues involved in tetralone interactions in the CYP26A1 active site (top ranked structures from Autodock). (**A**) tetralone 1; (**B**) tetralone 2; (**C**) tetralone 3; (**D**) tetralone 4; (**E**) tetralone 5. Ligand carbon atoms are colored in magenta. The tetralone—heme iron distances are in angstroms. Arg90 is colored yellow for orientation purposes only.

Molecular docking of the azoles R116010, R115866, liarozole, fluconazole, and five tetralone inhibitors was performed into the active site of CYP26A1, and their binding modes investigated after energy minimization. The CYP26A1 azole inhibitors show similar binding modes in which they occupy the active site of *at*RA with the azole ring oriented towards the heme iron, and are stabilized in the active site by interacting with several hydrophobic residues (Figure 7). The tetralones were also successfully docked in the enzyme active site and show hydrophobic interactions similar to *at*RA and the azoles (Figure 8).

Azole inhibitors block the catalytic cycle by coordinating to the CYP heme iron and thereby preventing oxygen binding and hydroxylation of the natural or exogenous substrates [42]. The azole nitrogen—heme iron distances obtained herein were all shorter than 3 Å (Table 1) which agrees well with values predicted from a previous density functional theory study of the corresponding distances between azoles and CYPs [42]. They are furthermore significantly shorter than that of *at*RA C4—heme iron (4.16 Å), giving further support for the current model in showing that the azoles efficiently block the access to the heme iron. Although the shortest distance between tetralones and the heme iron is larger than 3 Å, all distances are still shorter than the observed one for *at*RA, with the exception of tetralone 2 (5.82 Å). This result might explain that the weakest experimental inhibitory activity (30 µM) was indeed observed for this particular tetralone. Using a similar argument, the obtained distances indicate that the tetralones may inhibit *at*RA metabolism to a lesser extent than the azoles, which is also in agreement with experimental findings [24,28].

### 2.4. Binding Energy Calculations

Ligand binding energies were calculated after energy minimization and compared to experimental IC_50_ data from recent work by Thatcher *et al.* [28] and and Yee *et al.* [24] (Table 1). The calculated energies are in good agreement with experimental data except for tetralone **5**, which shows a slightly higher binding energy than expected considering the IC_50_ values. This deviation can be attributed to the presence of two hydroxyl groups which may result in an overestimation of the interaction between the molecule and the enzyme. Since the experimental IC_50_ values were obtained from different assays, it is not appropriate to compare the values for azoles and tetralones. However, the obtained binding energies show that the model can correctly distinguish between the natural substrate (inducer), strong inhibitors (R115866 and R116010) and weak inhibitors (liarozole, fluconazole, and tetralones).

## 3. Materials and Methods

### 3.1. Computational Approaches

All molecular modeling was performed using the YASARA Structure program [43]. Ligands, free CYP26A1 model, and ligand-model complexes were energy minimized to within an rms gradient of 0.1 kcal·mol^−1^·Å^−1^ using the Amber ff03 molecular mechanics force field [44]. In preparation for molecular dynamics simulations, the CYP26A1 model was solvated in a box of water followed by simulated annealing minimization. Taking pKa values into account, protonation states were assigned at physiologic pH 7.4 [45]. Water molecules were randomly replaced by sodium or chloride ions to achieve physiological salt concentration, and the solvent density was adjusted to 0.997 g·L^−1^, followed by Molecular Dynamics (MD) simulation and final energy minimization step. The MD simulation for the free CYP26A1 model was performed for 10 ns using periodic boundary conditions and the NVT canonical ensemble at 298 K. The simulation was performed in multiple time steps, 1.25 fs for intramolecular forces and 2.5 fs for intermolecular ones. The particle mesh Ewald (PME) method with a cutoff of 7.86 Å was used for long-range electrostatics [46]. Trajectory snapshots were sampled every 100 ps during the MD simulation.

### 3.2. Model Building

The fasta file of the 497 amino acid CYP26A1 sequence was obtained from NCBI protein (GI: 2688846). A prewritten macro in the YASARA program was used for model building, including sequence alignment, secondary structure prediction, threading, loop modeling and model refinement, using the crystal structure of the retinoic acid competent cyanobacterial CYP120A1 (PDB code 2VE3) [27] as template [47]. The initial sequence alignment of the target (CYP26A1) and template (CYP120A1) is shown in Figure S2 (Supplementary Materials).

### 3.3. Model Quality Assessment

The CYP26A1 model was carefully examined using three different online protein and homology model quality evaluation servers. A Ramachandran φ and ψ dihedral angles plot generated by the RAMPAGE server [48] was used to evaluate the stereochemical quality of the amino acids, folding reliability was assessed through the Verify3D structure server [49], and the model absolute quality based on six descriptive parameters was assessed using the QMEAN z-score server [50].

### 3.4. Ligands

The 3D structures of *at*RA and azole ligands were obtained from the ChemSpider database provided by the Royal Society of Chemistry [51]. The structures obtained from the database were the natural substrate *at*RA (ID 392618), and the azoles R115866 (ID 23281639), R116010 (ID 8046028), liarozole (ID 54664), fluconazole (ID 3248), and ketoconazole (ID 401695) (Figure 5). Tetralone derivatives structures were built using MOE’s program builder function as presented in our previous work where they were used to evaluate a CYP26B1 homology model [35,52].

### 3.5. Docking

A small grid of 30 × 30 × 30 points with a spacing of 0.375 Å was set around the model heme iron. Ligands were docked into the enzyme active site using AutoDock with a Lamarckian genetic algorithm [53] as embedded in YASARA. In the docking studies, flexible ligand conformations were generated by Monte Carlo methods while the receptor was held fixed. Flexible docking with integrated AutoDock is currently effective for ligands with up to 10 torsional degrees of freedom, this being the case for all studied ligands [43]. This approach in YASARA has proven to be very suitable to perform docking simulations [54,55,56]. In each docking round, a total of 25 independent docking runs were executed, retaining the lowest energy structure for each. The final docked conformations were categorized in clusters using an rmsd cutoff of 1 Å. The cluster with the best reported AutoDock binding energy was then selected for further consideration.

### 3.6. Binding Energy Calculations

In addition to the initial binding energies of the ligands as obtained by AutoDock (results not shown), the results were improved by calculating the binding energy in YASARA after energy minimization of the complexes in explicit water. The binding energy was obtained by calculating the energy at infinite distance between the ligand and the enzyme (*i.e.*, the unbound state) and subtracting the energy of the complex (the bound state). That is, a more positive binding energy indicates a more favorable interaction in the context of the force field used [43]. These energies are potential force field energies, not free energies. Thus, the binding energy does not include solvation or entropy contribution to the energy.

## 4. Conclusions

In this work, we presented an improved CYP26A1 homology model based on the retinoic acid bound cyanobacterial CYP120A1 structure. Docking of the natural CYP26A1 substrate (*at*RA) and a group of azoles and tetralones with known CYP26A1 inhibitor capability was successfully performed for all ligands (except ketoconazole). The distances between the relevant ligand atoms and the heme iron imply that the model is capable to differentiate structurally between natural substrate and inhibitors. The trends in calculated binding energies are also in agreement with recently reported experimental data, and show that the model is able to discriminate between natural substrate, strong inhibitors (R115866 and R1160110), and weak inhibitors (liarozole, fluconazole, and tetralones). The binding position of docked *at*RA suggests that (4*S*)-OH-*at*RA is the likely metabolite, which agrees with recent experimental data. We conclude that the model presented herein is reliable and has been improved over previously presented CYP26A1 models in terms of 3D structure and stereochemistry, and therefore can be further used in drug design campaigns or virtual screening search for new and improved RAMBAs.

## Figures and Tables

**Table 1:** CYP26A1 docked *at*RA, azole, and tetralone binding energies and distances from heme iron.

Inhibitor	IC_50_ (µM)	Binding Energy (kcal·mol^−1^)	Distance to Heme Iron (Å)
*at*RA (substrate)	Inducer	362	4.16
R115866	0.0043 ^1^	102	2.97
R116010	0.0051 ^1^	95	2.93
liarozole	2.1 ^1^	63	2.98
fluconazole	≥10 ^1^	60	2.96
tetralone 1	9 ^2^	50	3.51
tetralone 2	30 ^2^	48	5.82
tetralone 3	7 ^2^	51	3.66
tetralone 4	5 ^2^	56	3.40
tetralone 5	9 ^2^	62	3.62

^1^ Values obtained from CYP26A1 microsomal inhibition assay developed by Thatcher *et al.* [28]; ^2^ Values obtained from MCF-7 CYP26A1 cell assay by Yee *et al.* [24].

## Supplementary Materials

Supplementary materials can be accessed at: http://www.mdpi.com/1420-3049/21/3/351/s1.
