# Supplementary material for: Improved Homology Model of the Human all-trans Retinoic Acid Metabolizing Enzyme CYP26A1
Source: Molecules. 2016 Mar 15;21(3):351. doi: 10.3390/molecules21030351 (PMC6274249; doi:10.3390/molecules21030351)
Supplement: Supplementary file 1 [file molecules-21-00351-s001.pdf]

Supplementary Materials: Improved Homology Model of Human *all*-trans Retinoic Acid Metabolizing Enzyme CYP26A1

Mohamed K. A. Awadalla, Thamir M. Alshammari, Leif A. Eriksson and Patricia Saenz-Méndez

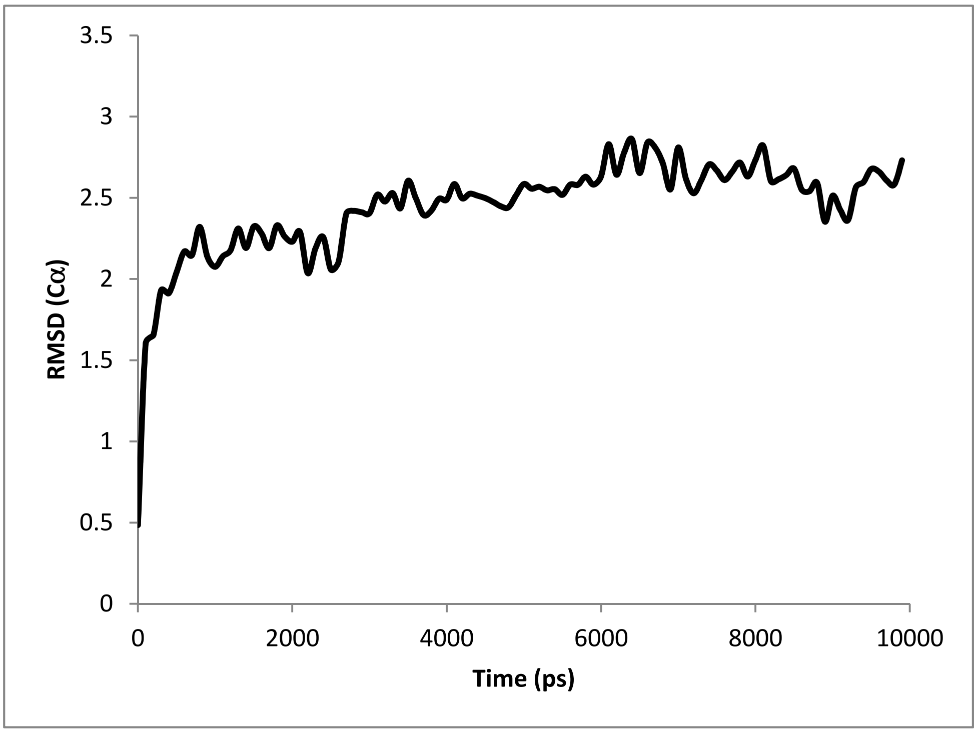

Figure S1. RMSD values of the positions of the Cα atoms versus time, of the homology model obtained.

```
Query  41   ALPLPPGTMGFPFFGETLQMVLQRRKFLQMKRRKYGFIYKTHLFGRPTVRVMGADNVRRI  100
            +LP+PPG  G P+ GETL   L    F + +++++G I+KT LFG+  + + GA   R +
Sbjct  10   SLPIPPGDFGLPWLGETLNF-LNDGDFGKKRQQQFGPIFKTRLFGKNVIFISGALANRFL  68

Query  101  LLGEHRLVSVHWPASVRTILGSGCLSNLHDSSHKQRKKVIMRAFSREALECYVPVITEEV  160
               E       WP S R +LG   L+       H+ R+K++ +AF    L+ Y+P +   V
Sbjct  69   FTKEQETFQATWPLSTRILLGPNALATQMGEIHRSRRKILYQAFLRPTLDSYLPKMDGIV  128

Query  161  GSSLEQWLSCGERGLLVYPEVKRLMFRIAMRILLGCEPQLAGDGDSEQQLVEAFEEMTRN  220
               LEQW    E  ++ YP+++R+ F +A  + +G +           QL   FE    +
Sbjct  129  QGYLEQWGKANE--VIWYPQLRRMTFDVAATLFMGEK------VSQNPQLFPWFETYIQG  180

Query  221  LFSLPIDVPFSGLYRGMKARNLIHARIEQNIRAKICGLRASEAGQGCKDALQLLIEHSWE  280
            LFSLPI +P +   +  +AR L+ A +E+ I+A+      +     +DAL +L+    +
Sbjct  181  LFSLPIPLPNTLFGKSQRARALLLAELEKIIKAR------QQQPPSEEDALGILLAARDD  234

Query  281  RGERLDMQALKQSSTELLFGGHETTASAATSLITYLGLYPHVLQKVREELKSKGLLCKSN  340
              + L +  LK     LLF GHET  SA +S    LG +  + ++VR+E     L
Sbjct  235  NNQPLSLPELKDQILLLLFAGHETLTSALSSFCLLLGQHSDIRERVRQEQNKLQL-----  289

Query  341  QDNKLDMEILEQLKYIGCVIKETLRLNPPVPGGFRVALKTFELNGYQIPKGWNVIYSICD  400
               +L  E L+++ Y+  V++E LRL PPV GGFR  ++  +  G+  PKGW V Y I
Sbjct  290  -SQELTAETLKKMPYLDQVLQEVLRLIPPVGGGFRELIQDCQFQGFHFPKGWLVSYQISQ  348

Query  401  THDVAEIFTNKEEFNPDRFMLPHPEDASR----FSFIPFGGGLRSCVGKEFAKILLKIFT  456
            TH   +++ + E+F+P+RF    P+ ++     F+ +PFGGGLR C+GKEFA++ +K+F
Sbjct  349  THADPDLYPDPEKFDPERFT---PDGSATHNPPFAHVPFGGGLRECLGKEFARLEMKLFA  405

Query  457  VELARHCDWQLLNGPP-TMKTSPTVYPVDNL  486
              L +  DW LL G    +  +P+  P DNL
Sbjct  406  TRLIQQFDWTLLPGQNLELVVTPSPRPKDNL  436
```

Figure S2. CYP26A1 (Query) and CYP120A1 (Template; "Sbjct") sequence alignment.
